# Single-Group Trial of an Internet-Delivered Insomnia Intervention Among Higher-Intensity Family Caregivers: Rationale and Protocol for a Mixed Methods Study

**DOI:** 10.2196/34792

**Published:** 2022-01-12

**Authors:** Kelly M Shaffer, Lee M Ritterband, Wen You, Daniel J Buysse, Meghan K Mattos, Fabian Camacho, Jillian V Glazer, Julie Klinger, Heidi Donovan

**Affiliations:** 1 Center for Behavioral Health and Technology University of Virginia Charlottesville, VA United States; 2 Department of Public Health Sciences University of Virginia Charlottesville, VA United States; 3 School of Medicine University of Pittsburgh Pittsburgh, PA United States; 4 School of Nursing University of Virginia Charlottesville, VA United States; 5 National Center on Family Support University of Pittsburgh Pittsburgh, PA United States; 6 School of Nursing University of Pittsburgh Pittsburgh, PA United States

**Keywords:** family caregiver, cognitive behavioral therapy, insomnia, sleep initiation and maintenance disorders, eHealth, protocol, mobile phone

## Abstract

**Background:**

Family caregivers are more likely to experience insomnia relative to noncaregivers but have significant barriers to accessing gold standard cognitive behavioral therapy for insomnia treatment. Delivering interventions to caregivers through the internet may help increase access to care, particularly among higher-intensity caregivers who provide assistance with multiple care tasks over many hours per week. Although there are existing internet interventions that have been thoroughly studied and demonstrated as effective in the general population, the extent to which these interventions may be effective for caregivers without tailoring to address this population’s unique psychosocial needs has not been studied.

**Objective:**

The goal of this trial is to determine what tailoring may be necessary for which caregivers to ensure they receive optimal benefit from an existing evidence-based, internet-delivered cognitive behavioral therapy for insomnia program named Sleep Healthy Using the Internet (SHUTi). Specifically, we will test the association between caregivers’ engagement with SHUTi and their caregiving context characteristics (ie, caregiving strain, self-efficacy, and guilt) and environment (ie, proximity to care recipient; functional status, cognitive status, and problem behavior of care recipient; and type of care provided). Among caregivers using the program, we will also test the associations between change in known treatment mechanisms (sleep beliefs and sleep locus of control) and caregiving context factors.

**Methods:**

A total of 100 higher-intensity caregivers with significant insomnia symptoms will be recruited from across the United States to receive access to SHUTi in an open-label trial with mixed methods preassessments and postassessments. At postassessment (9 weeks following preassessment completion), participants will be categorized according to their engagement with the program (nonusers, incomplete users, or complete users). Study analyses will address 3 specific aims: to examine the association between caregivers’ engagement with SHUTi and their caregiving context (aim 1a); to describe caregivers’ barriers to and motivations for SHUTi engagement from open-ended survey responses (aim 1b); and among caregivers using SHUTi, to determine whether cognitive mechanisms of change targeted by SHUTi are associated with differences in caregiving context (aim 2).

**Results:**

Institutional review board approvals have been received. Data collection is anticipated to begin in December 2021 and is expected to be completed in 2023.

**Conclusions:**

Findings will inform the next research steps for tailoring and testing SHUTi for optimal impact and reach among caregivers. Beyond implication to the SHUTi program, the findings will be translatable across intervention programs and will hold significant promise to reduce inefficiencies in developing digital health interventions for caregivers while also increasing their impact and reach for this underserved population.

**Trial Registration:**

ClinicalTrials.gov; NCT04986904; https://clinicaltrials.gov/ct2/show/NCT04986904?term=NCT04986904

**International Registered Report Identifier (IRRID):**

PRR1-10.2196/34792

## Introduction

### Background

An estimated 47.9 million Americans provide unpaid care to ≥1 family members or close individuals with serious health conditions [1]. Support from family members to individuals who are seriously ill is critical to the sustainability of the US health care system [2]; however, it places a significant strain on these caregivers. Insomnia is among the most common, distressing, and impairing psychophysiological issues for caregivers [3,4]. As the responsibilities and stressors of the caregiving role can both precipitate and perpetuate insomnia [5], caregivers are up to 3 times more likely to report sleep disturbances than the general population (up to 90% vs approximately 33%-50%, respectively) [6,7]. Cognitive behavioral therapy for insomnia (CBT-I), the gold standard treatment for insomnia [6], has shown promise among caregivers; however, their uptake and completion of this therapy has been limited [8-10]. As such, directly assessing how the caregiving context affects CBT-I engagement and efficacy could help to ensure that caregivers have equitable access to and benefit from this evidence-based intervention.

Among caregivers, those who spend many hours per week supporting multiple care tasks for a loved one—or *higher-intensity caregivers*—have more difficulty in accessing affordable support services, although they are also more interested in receiving support to manage their own emotional and physical well-being [1,11]. Existing psychosocial services for caregivers are primarily delivered in person. Although these interventions have generally been effective, they have low enrollment, high dropout, and limited reach to caregivers who already have inadequate health care access [12-15].

Digital health interventions can lower the barriers to entry to supportive care for higher-intensity caregivers, as they are conveniently accessible anywhere and anytime through an internet-enabled device. These interventions are also more scalable and sustainable than standard in-person practices [16]. For these reasons, caregivers themselves express strong interest in digital health interventions [17-19], and leaders in caregiving research have deemed the development and distribution of technology-based solutions to support caregivers as a high priority [20-23].

Most digital health interventions tested among caregivers have been developed de novo for specific caregiving contexts [24-29] in recognition of the unique deficits, risk factors, and needs that caregivers experience. Compared with noncaregivers, caregivers report unique barriers to broadly accessing psychosocial support, including guilt [30-32] and chaotic schedules [31,32]. More specifically, with regards to behavioral treatment for sleep, caregivers report unique worry about the impact of sleep loss on their ability to provide care [33-35] and challenges with nighttime symptom management [34,35]. Tailoring interventions to specific user groups such as caregivers increases information salience, which increases users’ attention to information and, therefore, the likelihood that the information will motivate behavior change [36]. When tested empirically, tailoring typically adds only small gains in outcomes relative to generic materials, although gains are typically maintained over time [37,38]. Although potentially appealing, tailoring reduces intervention reach as it narrows the user base and also increases the time for dissemination and costs for intervention development. Ultimately, the decision to tailor, and how to tailor, must balance any expected gains in treatment outcomes against the drawbacks of reduced reach and increased costs to maximize intervention impact.

Sleep Healthy Using the Internet (SHUTi [39,40]) is a fully automated, internet-delivered CBT-I program developed for the general population that holds significant promise for addressing insomnia among caregivers. In a recent trial of SHUTi among older adults, participants who self-identified as family caregivers (n=18) reported less improvement in their insomnia after using SHUTi (insomnia severity index [ISI] score difference=2.37, 95% CI 0.17-4.57; P=.03) and were less likely to rate their sleep quality as improved (*χ*^2^_1_ [N=190]=4.8; P=.03) compared with noncaregivers (n=189) [41]. However, significant differences (P>.10) between caregivers and noncaregivers were not observed for changes in cognitive mechanisms—more adaptive sleep beliefs and internalized sleep locus of control—or for ratings of program satisfaction, fit, or usability. Building on these preliminary observations, the goal of this trial is to determine what tailoring may be necessary for caregivers to receive optimal benefit from SHUTi.

To address this question, higher-intensity caregivers with significant insomnia symptoms will be recruited to receive access to SHUTi in a single-group, open-label trial with mixed methods preassessments and postassessments. This study design is recommended for establishing the plausibility of supporting subsequent fully powered efficacy testing [42]. At the end of the intervention period, caregivers will be categorized according to their level of engagement with the 6 SHUTi intervention lessons (or weekly *Cores*): nonusers (ie, completed 0 Cores), incomplete users (ie, completed ≤3 Cores), and complete users (ie, completed ≥4 Cores).

### Primary Trial Aims

#### Test the Association of SHUTi Engagement With Caregiving Context

Specifically, we will test how caregivers’ engagement with SHUTi is associated with their user characteristics and environmental characteristics (aim 1a). We will also describe caregivers’ barriers to and motivations for SHUTi engagement from open-ended survey responses (aim 1b).

#### Test the Association Between SHUTi Efficacy on Known Cognitive Mechanisms With Caregiving Context

Among caregivers using SHUTi, we will test whether cognitive mechanisms of change targeted by SHUTi are associated with differences in caregiving-related user or environmental characteristics.

## Methods

### Participants

This protocol manuscript has been developed in accordance with reporting recommended by the 2013 SPIRIT (Standard Protocol Items: Recommendations for Interventional Trials) statement [43,44]; see Textbox 1 for key study information. The protocol was revised following peer review through the National Institutes of Health; see Multimedia Appendix 1 for reviews and Multimedia Appendix 2 [1,6,40,45-54] for our team’s response to reviews during the *just in time* period for our responsive protocol changes. Participants will be higher-intensity family caregivers reporting significant insomnia symptoms. Family caregiving is defined according to the National Alliance for Caregiving 2020 Caregiving in the United States survey methods [1]. Specifically, a family caregiver will be someone who is currently providing unpaid care to a relative or friend, aged ≥18 years, to help them take care of themselves and/or providing more than the normal amount of unpaid care for a child aged <18 years because of a medical, behavioral, or other condition or disability.

Higher-intensity caregiving will be defined by scoring ≥5 points on a scale modified from the National Alliance for Caregiving Level of Care Index. Scoring is the function of the given number of hours of care and types of care tasks provided for activities of daily living (ADL; including medical and nursing tasks), instrumental ADL, and for child care recipients, selected caregiver support activities. If a caregiver provides care to >1 care recipient, the total amount of time and all care tasks across all care recipients are considered together to compute the care intensity score. See Table 1 for point assignments, which are summed to a total index score.

Although we considered reducing sample heterogeneity in the caregiving context, we chose to keep the caregiving inclusion criteria broad as it increases the generalizability of our findings. A broad sample will also facilitate our aim of understanding how broad or specific a caregiving audience can be addressed by a particular digital health intervention. Higher-intensity caregivers, regardless of disease context, share significant psychological (eg, guilt) and practical (eg, limited time) barriers that make them less likely than lower-intensity caregivers to be able to access care while also at risk for worse health outcomes. Therefore, our sample of higher-intensity caregivers will provide actionable insights into how to increase digital health intervention access to caregivers with the most barriers to care.

Potentially eligible caregivers must also have regular access to the internet (whether by computer, tablet, or smartphone) and be willing to receive study-related emails. Internet access is required to complete the SHUTi intervention. Although this requirement may introduce some bias to the sample, there is a strong rationale for it. Using 2017 nationally representative survey data [55], we found that 88% of caregivers reported accessing the internet (vs 89% of the general population [56]), and there was no difference in internet access by caregivers’ level of distress, burden, or rurality [57]. Limitations in reach imposed by requiring internet access for this study will be offset by the increased convenience of a fully automated internet intervention (relative to the care delivered in person or scheduled with a provider).

Eligible caregivers will also be required to score ≥10 on the 7-item ISI [58]. This score corresponds to the cutoff suggestive of clinically significant insomnia symptoms among community samples [58]. All inclusion and exclusion criteria are listed in Textbox 1.

**World Health Organization trial registration data set.**

**Primary registry and trial identifying number**

- ClinicalTrials.gov NCT04986904

**Date of registration in primary registry**

- August 3, 2021

**Secondary identifying numbers**

- R21TR003522
- University of Virginia Health Sciences Research Institutional Review Board protocol HSR210255
- University of Pittsburgh Office of Research Protections Institutional Review Board protocol STUDY21080076

**Source of monetary or material support**

- National Institutes of Health–National Center for Advancing Translational Sciences (*this funding source has no significant role in the design of this study and will not have any role during its execution, analyses, interpretation of the data, or decision to submit the results*)

**Primary sponsor**

- University of Virginia

**Secondary sponsor**

- University of Pittsburgh

**Contact for public queries and scientific queries**

- Kelly M Shaffer, PhD—Center for Behavioral Health and Technology, University of Virginia, Charlottesville, United States of America; (434) 982-1022; kshaffer (at) virginia.edu

**Public title**

- SHUTi CARE (Sleep Healthy Using the Internet–caregiver acceptability research)

**Scientific title**

- Optimizing efficiency and impact of digital health interventions for caregivers: A mixed methods approach

**Country of recruitment**

- United States

**Health conditions or problems studied**

- Family caregivers
- Insomnia
- Sleep initiation and maintenance disorders

**Intervention**

- SHUTi—internet-delivered cognitive behavioral therapy for insomnia program

**Ages eligible for study**

- ≥18 years

**Sexes eligible for study**

- All

**Accepts healthy volunteers**

- No

**Inclusion criteria**

- Caregiving
  - Current higher-intensity caregiving (ie, level ≥3 on National Alliance for Caregiving Level of Care index)
  - Expect to provide high-intensity caregiving for at least another 3 months (study duration)
- Sleep
  - Insomnia severity index score ≥10
- Internet access
  - Have access to any internet-enabled device (computer, tablet, or smartphone)
  - Willing to be emailed about the study
- Miscellaneous
  - Able to speak and read English
  - Residing in the United States or US territory

**Exclusion criteria**

- Sleep
  - Irregular schedule that would prevent adoption of intervention strategies (ie, shift work and typical bedtime earlier than 8 PM or later than 2 AM or arising time earlier than 4 AM or later than 10 AM)
  - Current behavioral or psychological treatment for insomnia
- Medical and psychiatric contraindications
  - Presence of another unmanaged sleep disorder (restless leg syndrome or periodic limb movement disorder, obstructive sleep apnea, narcolepsy, or parasomnia)
  - Diagnosis of dementia, Alzheimer disease, Parkinson disease, Huntington disease, schizophrenia, or psychosis
  - History of the following without recovery: stroke, traumatic brain injury, brain infection, or brain tumor
  - Current pregnancy or breastfeeding, chemotherapy for cancer, alcohol dependence or abuse, or substance dependence or abuse
  - Current unmanaged hyperthyroidism, severe respiratory disease, or epilepsy
  - Change in medication regimen for steroids, amphetamines stimulants, or prescribed sleep medications within the past 3 months
  - History of manic or hypomanic episode
- Miscellaneous
  - Severe computer literacy challenges

**Study type**

- Interventional

**Allocation**

- Not applicable

**Intervention model**

- Single-group assignment

**Masking**

- None (open label)

**Primary purpose**

- Treatment

**Date of first enrollment**

- December 2021 (anticipated)

**Target sample size**

- 100

**Recruitment status**

- Recruiting

**Primary outcome**

- SHUTi engagement (time frame: 9-week postassessment)

**Key secondary outcomes**

- Open-ended feedback about SHUTi (time frame: 9-week postassessment)
- SHUTi evaluation (time frame: 9-week postassessment)
- Change in sleep-related cognitions (time frame: preassessment and 9-week postassessment)
- Change in sleep locus of control (time frame: preassessment and 9-week postassessment)

**Table 1:** Level of care index for higher-intensity caregiving^a^.

Points	Hours of care (average week)	Type of care provided	
		Adult care recipient	Child care recipient^b^
1	0-8	0 ADL^c,^^d^, 1 IADL^e^	0 ADL, 1 IADL or CSA^f^
2	9-20	0 ADL, ≥2 IADLs	0 ADL, ≥2 IADL or CSAs (eg, 1 IADL plus 1 CSA)
3	21-40	1 ADL, any number IADLs	1 ADL, any number IADL or CSAs
4	≥41	≥2 ADLs, any number IADLs	≥2 ADLs, any number IADL or CSAs

^a^On the basis of the National Association for Caregiving (2020) Level of Care Index.

^b^Caregivers for child care recipients are only asked about developmentally appropriate ADLs and 3 IADLs: assisting with medical or nursing tasks, managing finances, and arranging outside services.

^c^ADL: activity of daily living.

^d^Includes medical and nursing tasks.

^e^IADL: instrumental activity of daily living.

^f^CSA: caregiver support activities—advocating with health care providers, community services, schools, or government agencies; monitoring the severity of their condition; communicating with health care professionals.

### Procedures

**Figure 1:**
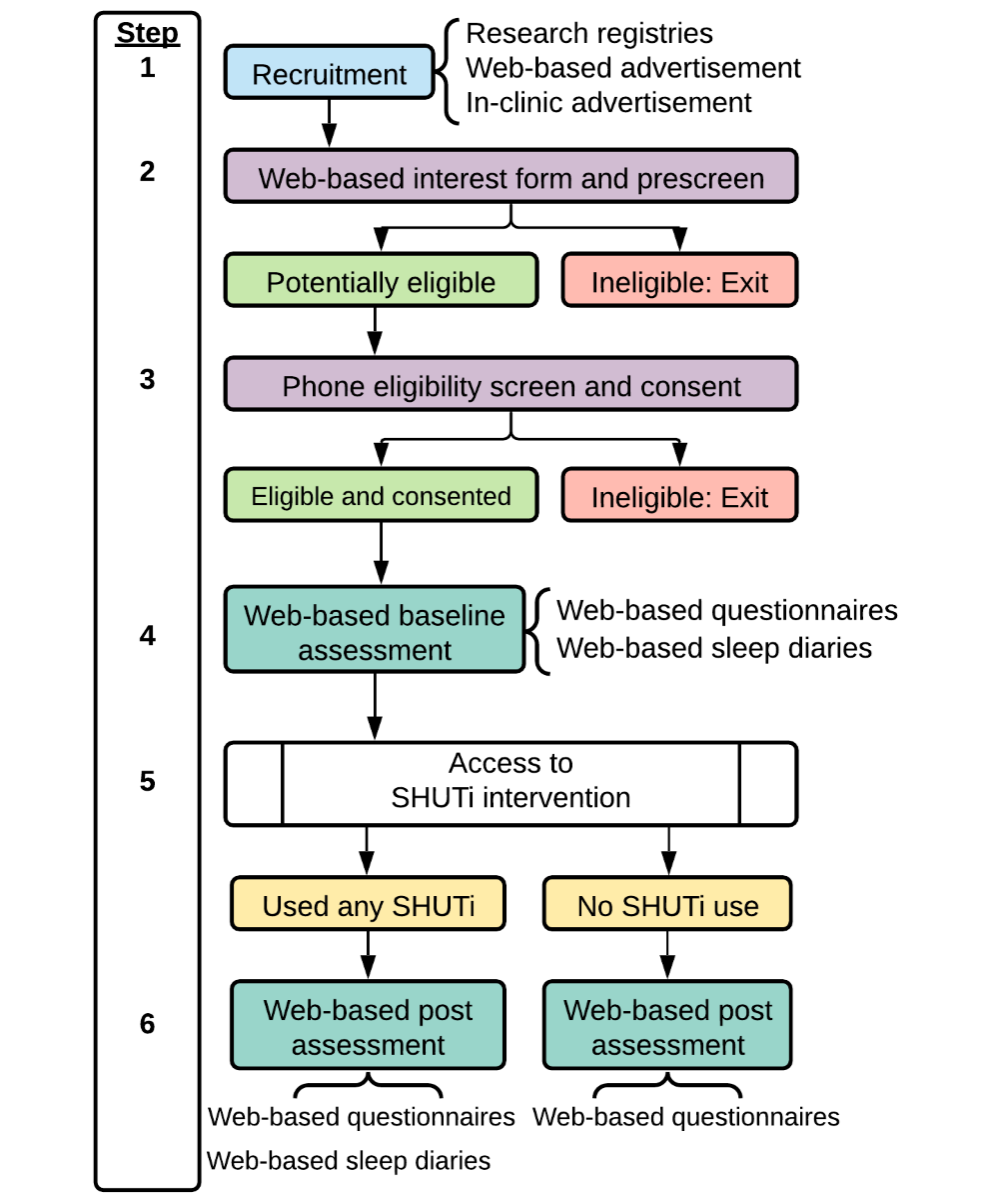
Trial procedures. SHUTi: Sleep Healthy Using the Internet.

Potential participants will learn about our trial from any of the three primary recruitment pathways (Figure 1 step 1): (1) University of Pittsburgh research registries, either receiving a letter through the Center for Social and Urban Research Caregiver Research Registry or matching with the study through the Clinical and Translational Science Institute Pitt+Me Research Registry; (2) web-based advertisement, including national social media advertisement campaigns by the University of Virginia Health System, social media postings, and website and newsletter postings through pertinent community organizations; or (3) in-clinic advertisements and informational flyers and handouts provided through partnering clinics at the University of Virginia and University of Pittsburgh.

Potentially interested individuals will contact the research staff and visit the study website for more information about the study. Interested individuals will complete a brief web-based interest and prescreening form (step 2). Where indicated, individuals’ identities will be verified using TLOxp (TransUnion), a web-based people search tool [59]. Potentially eligible individuals will be contacted by research staff by phone to complete eligibility screening, answer any remaining questions about the trial, and obtain informed consent to participate (step 3). Informed consent to participate will be collected electronically via DocuSign, and participants will have the opportunity to download a digitally signed copy of the form.

Enrolled participants will then be emailed log-in information for the SHUTi intervention website to complete preassessment (step 4). They will first complete a web-based questionnaire battery; then, participants will complete daily web-based sleep diaries through the SHUTi system. Participants must enter 10 daily sleep diaries in 14 days to advance through this stage. Upon completing these preassessment sleep diaries, participants will be compensated US $40.

Following completion of the preassessment, all participants will be advanced to the SHUTi intervention in this single-group, open-label trial (step 5; see *Intervention* section for more details). At the end of the 9-week intervention period, regardless of intervention progress, participants will complete postassessment (step 6). If a participant completes ≥1 Core, they will complete the full battery of questionnaires and 10 web-based sleep diaries. If a participant completes no Cores, they will complete a brief postassessment questionnaire battery. These nonusers will not be asked to complete the full battery of questionnaires or sleep diaries to encourage retention. Participants will be compensated US $40 upon completion of the postassessment.

### Intervention

Our team has established SHUTi as an effective treatment for insomnia over the past 15 years [39,40,45,60-65]. A complete description of the intervention has been published previously [40]; key intervention details are included here. SHUTi is a fully automated internet-delivered insomnia intervention that is tailored from user-inputted data. The intervention is based on CBT-I, covering the primary tenets of sleep restriction, stimulus control, cognitive restructuring, sleep hygiene, and relapse prevention [40,66]. Study staff will be available to provide technical support; however, no clinical support will be provided to users. In this trial, participants will receive access to the standard SHUTi program—meaning that the program has no tailoring to any specific user population. There is no content in the program that specifically addresses caregiving or caregiving-related impacts on sleep or treatment.

SHUTi content will be metered out over time through 6 *Cores*, or lessons, each of which will take approximately 45 minutes to 1 hour to complete. Core content (Textbox 2) will be delivered in a way that is designed to be engaging based on learning theory and instructional design [67], with interactive features such as graphical feedback of entered data, animations, quizzes, and videos. Cores will be delivered to users on a time- and activity-based schedule. Specifically, the next Core will be made available to the user 1 week following their completion of the prior Core, which will allow participants time to practice skills. Users can track their sleep using daily web-based sleep diaries, each of which will take approximately ≤3 minutes to complete. The intervention will provide tailored content to users based on these diaries (eg, tailored *sleep prescriptions* as part of the sleep restriction technique) and other user-entered data. Participants will receive regular automated emails from the SHUTi program to support user engagement (eg, reminders to complete sleep diaries and notifications that a new Core is available).

**Sleep Healthy Using the Internet intervention content by Core.**

**Core 1: Overview**

Insomnia defined, types, prevalence, risk factors, and impact (ie, daytime fatigue, psychological well-being, physical health, and economic cost); setting treatment goals; and treatment overview, appropriateness, and effectiveness

**Core 2: Sleep Behavior 1**

Explanation of poor sleep habits; situational or chronic sleep difficulties; cycle of chronic insomnia; introduction of sleep restriction; explanation of sleep efficiency; and instruction on adjustments of sleep window based on sleep efficiency

**Core 3: Sleep Behavior 2**

Introduction of stimulus control (ie, going to bed when sleepy, leaving bed if unable to sleep, regular sleep schedule, using bed for sleep only, and no napping)

**Core 4: Sleep Thoughts**

Relationship between thinking patterns and emotions; contributions of thought patterns to sleeplessness; cognitive restructuring; keeping realistic expectations; revising misconceptions about insomnia; eliminating catastrophizing; reducing sleep emphasis; developing tolerance for sleep loss effects; and dealing with setbacks

**Core 5: Sleep Education**

Sleep hygiene guidelines; avoiding stimulants; and effects of diet, environment, and exercise

**Core 6: Problem Prevention**

Relapse prevention techniques; considering therapeutic gains; review of sleep behavior techniques; sleep medication information; and maintaining program techniques

### Measures

#### Overview

Study measures are summarized in Table 2. Web-based questionnaires will be completed through a Health Insurance Portability and Accountability Act–compliant web-based survey manager (Qualtrics Highly Sensitive Data). All items will include validation to notify participants if they skip an item to reduce accidentally missing data; however, responses will not be required, as participants may skip any question they would like.

**Table 2:** Measures.

Variable and measure		Outcome measured	Time	
			Pre	Post
**Sample characteristics**				
	Sociodemographics	Age, gender, race or ethnicity, household income, health literacy, and relationship to care recipient	✓	
**Predictors: caregiving-related user characteristics**				
	Caregiving strain	Pearlin Stress Scale–caregiving overload subscale	✓	✓
	Caregiving self-efficacy	Pearlin Stress Scale–caregiving competence subscale	✓	
	Caregiving guilt	Caregiver Guilt Questionnaire–guilt about doing wrong by the CR^a^, not rising to the occasion as a caregiver, and self-care subscales	✓	
**Predictors: caregiving-related environmental characteristics**				
	Proximity to CR^a^	Whether bedpartner, live together but not bedpartner, or other situations	✓	
	CR functional status	Modified Barthel Activities of Daily Living Index	✓	
	CR cognitive status	Pearlin Stress Scale–cognitive status subscale	✓	
	CR problem behavior	Pearlin Stress Scale–problematic behavior subscale (includes nighttime problems)	✓	
	Caregiving tasks	Involvement in supporting activities of daily living, instrumental activities of daily living, and caregiver support activities	✓	
	Changes in caregiving	Single item question (with follow-up open-ended response) to assess if caregiving situation has significantly changed during study		✓
**Aim 1 outcomes: SHUTi^b^ engagement**				
	Core completion	Nonuser (no Cores completed); incompleter (1-3 Cores); completer (4-6 Cores)	Throughout SHUTi	
	Open-ended feedback	Free-response survey items—separate surveys for nonusers and users		✓
	SHUTi utility and barriers	Internet Intervention Utility, Evaluation, and Adherence Questionnaires—selected items		✓^c^
**Aim 2 outcomes: SHUTi efficacy on known cognitive mechanisms**				
	Sleep beliefs	Dysfunctional Beliefs and Attitudes About Sleep	✓	✓^c^
	Sleep control	Sleep Locus of Control Scale	✓	✓^c^
**Exploratory: preliminary efficacy**				
	Insomnia severity	Insomnia severity index-2 item	✓	✓
	Sleep diary metrics	10 days of sleep diaries in 14-day period	✓	✓^c^
	Health-related quality of life	PROMIS^d^: 2-item Global Physical Health	✓	✓
	General distress	Patient Health Questionnaire-4	✓	✓

^a^CR: care recipient.

^b^SHUTi: Sleep Healthy Using the Internet.

^c^Assessed among SHUTi users (ie, incompleters and completers) only.

^d^PROMIS: Patient-Reported Outcomes Measurement Information System.

#### Outcomes: Aim 1

The primary trial outcome will be the level of participants’ engagement with SHUTi, operationalized by intervention Core completion. Participants will be categorized according to their Core completion: (1) nonusers, who complete no Cores; (2) incompleters, who complete 1 to 3 Cores; and (3) completers, who complete 4 to 6 Cores. Those completing ≥4 Cores are considered to have completed the program as they have completed content related to primary treatment change mechanisms; also, approximately all (>90%) participants continue on to complete all 6 Cores based on multiple past SHUTi trials. Participants’ Core completion will be automatically tracked by the SHUTi intervention platform.

Following the intervention period, we will also assess caregivers’ barriers to the uptake and use of SHUTi (nonusers); their perceived satisfaction, utility, and efficacy of SHUTi (users—both incompleters and completers); and ways in which we may tailor the SHUTi program to better fit family caregivers’ needs related to sleep. Depending on whether the participant is a nonuser or user, they will complete open-ended free-response items, 4 items from the Client Satisfaction Questionnaire [68], and selected items from the Internet Intervention Utility, Evaluation, and Adherence Questionnaires [40].

#### Outcomes: Aim 2

We will assess SHUTi users’ change in key cognitive mechanisms of sleep beliefs (Dysfunctional Beliefs and Attitudes about Sleep [69]) and sleep locus of control (Sleep Locus of Control Scale [70]). These variables have been previously demonstrated to mediate SHUTi benefits on insomnia symptom severity [45].

#### Predictors: Caregiving Context

We will test the association of SHUTi engagement and change in cognitive mechanisms with caregiving-related user characteristics and environmental characteristics.

##### Caregiving-Related User Characteristics

We will assess 3 potentially modifiable, subjective aspects of caregiving: (1) caregiving strain (Pearlin Stress Scale [PSS]–caregiving overload subscale [71]), (2) caregiving self-efficacy (PSS–caregiving competence subscale [71]), and (3) caregiving guilt (Caregiver Guilt Questionnaire—guilt about doing wrong by the care recipient, not rising to the occasion as a caregiver, and self-care subscales [72]).

##### Caregiving-Related Environmental Characteristics

We will also assess 5 structural factors in the caregiving situation. In the event that the caregiver provides care to >1 care recipient, they will respond to the following about their main care recipient (ie, care recipient to whom they provide the most care), as has been done previously [1]: (1) proximity to the care recipient—whether bedpartner, living together but not bedpartner, or other living situation—(2) care recipient’s functional status (Modified Barthel ADL Index [73]), (3) care recipient’s cognitive status (PSS–cognitive status subscale [71]), (4) care recipient’s problem behavior (PSS–problematic behavior subscale [71]), and (5) care provided—involvement in supporting ADL, instrumental ADL, and caregiver support activities.

#### Preliminary Efficacy Outcomes

As an exploratory analysis of SHUTi efficacy, we will also examine changes in caregivers’ self-reported insomnia severity (ISI-2 item [74]), metrics from sleep diaries (eg, sleep onset latency, wake after sleep onset, and sleep quality; SHUTi users only), health-related quality of life (Patient-Reported Outcomes Measurement Information System Global Physical Health-2 item [75]), general distress (Patient Health Questionnaire-4 [76]), and caregiving strain (PSS–caregiving overload subscale [71]).

### Sample Size and Power Analysis

A total of 100 caregivers will be enrolled in this trial. This sample size was identified based on a power analysis to detect moderate effects of the caregiving context variables on the primary outcome of engagement with SHUTi. We estimated the expected caregiver engagement with SHUTi based on our team’s prior research. On the basis of engagement rates from a recently completed trial of an untailored internet depression management program among family caregivers [77] (nonusers=35%; incompleters=40%; completers=25%) and average completion rates across multiple SHUTi trials [61-63] (nonusers=10%; incompleters=25%; completers=65%), we estimate that the sample in the proposed trial will be categorized as follows: 22% (22/100) nonusers, 33% (33/100) incompleters, and 45% (45/100) completers. Assuming these engagement rates, the minimally detectable proportional odds for aim 1a analyses at power=80% with α=.05 would be a moderate effect size [78] (minimally detectable proportional odds=2.9) for a sample size of N=100 based on the rate of dichotomized predictor exposures (computed using the Whitehead formula [79] in R Hmisc [80]). An example of a detectable scenario (user status associated with caregiving strain) under these conditions is presented in Table 3. In this hypothetical situation, caregivers who did not complete any SHUTi Cores (nonusers) were more likely to report high caregiving strain relative to caregivers who completed any SHUTi Cores (users). As this primary analysis uses preassessment questionnaire data, and engagement is automatically assessed in the SHUTi intervention platform, the final sample size determination does not need to account for attrition.

**Table 3:** Example of a detectable scenario for aim 1a (odds ratio 3.4; N=100).

Engagement status	Caregiving strain			Sample (%)
	Above median	Below median		
Nonuser, n (%)	16 (72.7)	6 (27.3)	22	
User, n (%)	34 (43.6)	44 (56.4)	78	
Sample (%)	50	50	100	

### Data Analysis Plan

#### Aim 1a: Test the Association of SHUTi Engagement With Caregiver User and Environmental Characteristics

To test the effect of caregiver context on SHUTi engagement, ordered logistic regressions assuming proportional odds will be fit for each caregiving-related user and environmental characteristics on SHUTi Core completion, which is a 3-level ordinal-dependent variable (ie, nonusers vs incompleters vs completers). Where the proportional odds assumption is violated (P<.05), follow-up sensitivity analyses will be conducted: 2 logistic regressions will compare proportional odds between nonusers and users (incompleters and completers) and between noncompleters (nonusers and incompleters) and completers. For users, we will also examine the associations between user and environmental characteristics with SHUTi evaluations on the Internet Intervention Utility, Evaluation, and Adherence Questionnaires.

#### Aim 1b: Describe Caregivers’ Barriers to and Motivations for SHUTi Engagement

Users’ open-ended survey responses from the postassessment will be qualitatively coded using 2 methods. First, an a priori codebook will be used to tag data according to whether it is (1) specific to SHUTi, (2) specific to CBT-I but not exclusively SHUTi, or (3) specific to digital health interventions but not exclusively SHUTi. This will facilitate the examination of the extent to which caregiver-specific tailoring recommendations are specific to SHUTi versus generalizable to other evidence-based psychosocial digital health interventions. Next, responses will be coded inductively using thematic text analysis [81] to identify themes related to caregivers’ barriers to and motivations for SHUTi uptake and use as well as how caregiver-specific tailoring may affect each of those constructs. The coding team will iteratively determine a set of codes; identify, review, and name themes; and synthesize data into final actionable recommendations for tailoring SHUTi and other digital health interventions for caregivers.

Findings and resulting recommendations will be returned to caregivers who indicate that they are willing to be recontacted for synthesized member checking [82]. A concise report of the results will be sent to the caregivers. Then, the caregivers will review the report and comment on how the results compare and contrast with their experiences and needs, and their returned responses will be coded to ascertain the level of resonance with the researchers’ results. The findings and recommendations will be revised according to the synthesized member checking results.

#### Aim 2: Test the Association of SHUTi Efficacy on Known Cognitive Mechanisms With Caregiving Context

Among SHUTi users, continuous regression modeling will test the association of each caregiving context predictor with cognitive mechanisms assessed at postassessment, controlling for preassessment [83]. Models will control for the level of SHUTi engagement, with significance set at α=.05.

#### Preliminary Efficacy

The preliminary efficacy of SHUTi for caregivers will be explored by computing within-group effect size on the change from preassessment to postassessment on insomnia symptoms (ie, self-reported severity), sleep outcomes (ie, sleep diary metrics), and related constructs of general distress and caregiving strain.

## Results

This study is funded by the National Institutes of Health–National Center for Advancing Translational Sciences (R21TR003522; project duration: July 2021-June 2023). The study protocol has been reviewed and approved by the University of Virginia (UVA) institutional review board (IRB) for health sciences research (protocol HSR210255; initial approval date: September 2021), which serves as the IRB of record. The protocol has also been initiated through the University of Pittsburgh Office of Research Protections (STUDY21080076). Study modifications will be reviewed and approved by the UVA IRB, and the University of Pittsburgh IRB will be notified of these modifications as necessary. The UVA IRB will review the protocol and progress reports for this trial annually. This trial is registered with ClinicalTrials.gov (NCT04986904). Data collection is anticipated to begin in December 2021 and is expected to be completed in 2023.

## Discussion

### Principal Findings

This study will provide the data necessary to ensure the highest impact and efficiency from existing evidence-based digital health interventions to meet pressing psychosocial needs among caregivers. Specifically, this study will inform the next research steps for tailoring and testing SHUTi for optimal impact and reach among caregivers. More broadly, our results will address a key translational science research question for the caregiving field, namely, how existing evidence-based digital health interventions can be most effectively translated to meet psychosocial needs among caregivers

This is the first study to address this broad translational question by systematically studying what tailoring may be necessary to increase the impact of an existing evidence-based digital health intervention program among higher-intensity caregivers. Much of the available psychosocial care for caregivers is delivered face to face, a modality that has significant practical and financial barriers limiting accessibility for caregivers [31,32]. The findings will help address how to serve caregivers more quickly and effectively by addressing prevalent and high-burden psychosocial concerns with existing evidence-based digital health interventions. As the effects of SHUTi have been robustly established across multiple clinical trials, the present trial among family caregivers will be able to isolate the effects of caregiving context on the use and impact of SHUTi for this specific population.

Relatedly, this trial takes a fundamentally different approach to caregiving intervention development by directly studying the ways in which key intervention targets should differ—or not—according to the caregiving context. Caregiver interventions have primarily been developed for specific caregivers defined by the care recipient’s disease [22,26,29]. This is appropriate for certain psychoeducational or caregiver training interventions. However, for interventions targeting specific problems such as insomnia, which is caused and exacerbated by multiple behavioral and psychological factors, it is not clear whether needs differ across caregiving contexts to justify increased specificity in the intervention. This trial will specifically assess how the caregiving context may relate to intervention engagement and efficacy among higher-intensity caregivers who are known to be at the highest risk for poor outcomes but have the most difficulty accessing interventions [1,11]. By examining what tailoring may be necessary for which caregivers, we can better balance intervention efficacy against reach by ensuring that interventions are applicable to the broadest possible population of family caregivers.

We expect that findings from this mixed methods proposal will direct the next research steps toward 1 of 2 primary paths. If the findings suggest that caregiving-related user and environmental characteristics are not related to SHUTi engagement or efficacy, with no significant intervention tailoring requirements identified by caregivers, then a trial designed to understand the efficacy of and dissemination strategies for the existing SHUTi program (without tailoring) among caregivers will be warranted. On the other hand, if findings suggest meaningful differences in SHUTi engagement or efficacy by caregiving context or if key themes arise regarding intervention tailoring that may better engage and support caregivers, then tailoring and testing SHUTi for caregivers will be warranted. Potential changes may include modifying content to be more caregiver salient, addressing specific caregiving-related barriers to sleep recommendations, or wrapping the intervention in an implementation intervention to promote engagement.

Findings from the proposed work will not only be necessary to direct the next research on SHUTi specifically but will also deliver key insights on tailoring other evidence-based digital health interventions for caregivers. By doing so, findings will advance the science toward our long-term goal of improving the quality and impact of digital health interventions for caregivers while reducing intervention development inefficiency—a goal identified as a high priority for current caregiving research. As such, findings will be translatable across intervention programs and hold significant promise for reducing inefficiencies in developing digital health interventions for caregivers while also increasing their impact and reach for this underserved population.

## Appendix

Multimedia Appendix 1 Peer-review report by the National Center for Advancing Translational Sciences (NCATS), National Institutes of Health (NIH, USA).

Multimedia Appendix 2 Response to National Institute of Health reviewer feedback submitted at just-in-time phase.

## Abbreviations

ADL: activities of daily living
CBT-I: cognitive behavioral therapy for insomnia
IRB: institutional review board
ISI: insomnia severity index
NIH: National Institutes of Health
PSS: Pearlin Stress Scale
SHUTi: Sleep Healthy Using the Internet
SPIRIT: Standard Protocol Items: Recommendations for Interventional Trials
UVA: University of Virginia
