# Supplementary material for: Single-Group Trial of an Internet-Delivered Insomnia Intervention Among Higher-Intensity Family Caregivers: Rationale and Protocol for a Mixed Methods Study
Source: JMIR Res Protoc. 2022 Jan 12;11(1):e34792. doi: 10.2196/34792 (PMC8792774; doi:10.2196/34792)
Supplement: Multimedia Appendix 1 [file resprot_v11i1e34792_app1.pdf]

1R21TR003522-01A1 Shaffer, Kelly

RESUME AND SUMMARY OF DISCUSSION: This is a resubmitted application from the University of Virginia for the Limited Competition: Clinical and Translational Science Award (CTSA) Program: Exploratory Collaborative Innovation Awards (R21 Clinical Trial Optional). This collaboration is between the University of Pittsburgh and the University of Virginia (UVA). This application aims to identify what is necessary to tailor and optimize the efficiency and acceptability of an insomnia digital tool, SHUTi, for use among caregivers. Strengths of the application include an important topic to improve digital health interventions that can greatly benefit caregivers with insomnia; tailoring of the application to a specific group; a plan to assess the needs of high intensity caregivers, and a strong and experienced team of investigators. The resubmission is responsive to the previous critiques and has been significantly changed to address prior concerns. There are some weaknesses that linger including lack of clarity regarding the six Cores of the tool, lack of justification for changing the age range of caregivers when the prior research has been done on people 55 years and older, and limited details on how the findings will inform the tailoring of the tool. In addition, details regarding the quantitative analysis are sparce; there are concerns over the amount of time needed to complete the measure for a group that is already burdened; and there are some concerns that an open-ended post assessment survey might not inform sufficiently. Overall, the application received an Impact/Priority Score of 22; the committee recommends the budget as requested.

DESCRIPTION (provided by applicant): One in six American adults provide care for a loved one with disabling illness, and these family caregivers are more likely to experience insomnia and other psychological concerns than the general population. Multiple existing, evidence-based digital health interventions may effectively address caregivers' psychosocial needs and increase caregivers' access to supportive care. For example, Sleep Healthy Using the Internet (SHUTi) developed by co-I Ritter band is an NCI-designated research-tested intervention that delivers cognitive- behavioral therapy for insomnia. A key translational research question remains about existing evidence-based digital health interventions like SHUTi, namely, what level of tailoring would be necessary and sufficient achieve optimal engagement with and efficacy of these interventions for caregivers? To address this research question, we will recruit 100 high-intensity caregivers with insomnia to complete a baseline assessment of insomnia and caregiving context. Caregivers will then receive access to SHUTi in an open-label trial, then complete post- assessment and be categorized according to their level of engagement with the 6 intervention "Cores": non- users (i.e., completed no Cores), incomplete users (i.e., 1 to 3 Cores), and complete users (i.e., 4 to 6 Cores). For Aim 1, we will test the association of SHUTi engagement with caregiving context. First, we will test whether caregivers' engagement with SHUTi (i.e., being a non-user vs. incomplete user vs. complete user) is associated with their user characteristics (i.e., caregiving strain, self-efficacy, and guilt) and environment characteristics (i.e., proximity to care recipient; care recipient functional, cognitive, and behavioral status; caregiving tasks; Aim 1a). Second, we will describe caregivers' barriers to and motivations for SHUTi engagement from their responses to open-ended surveys, and how caregiver-specific tailoring may improve uptake and usage (Aim 1b). Thematic coding will also examine how caregivers' recommendations generalize to other evidence-based digital health interventions, and findings will be validated using synthesized member checking. For Aim 2, we will test whether the effects of SHUTi on known cognitive mechanisms of change targeted by SHUTi (i.e., more adaptive sleep beliefs, internalized sleep locus of control) are associated with differences in caregiving-related user and environment characteristics. Findings from these two aims are not only necessary to direct next research on tailoring and testing SHUTi for caregivers specifically, but also to advance the science towards our long-term goal, namely, to improve the quality and impact of digital health interventions for caregivers, while reducing intervention development inefficiency – a goal identified as a high priority for current caregiving research. As such, findings will be translatable across research-tested intervention programs and hold significant promise to reduce inefficiencies in developing digital health interventions for caregivers, while also increasing intervention impact and reach for this underserved population.

PUBLIC HEALTH RELEVANCE (provided by applicant): The proposed research is relevant to public health because about one in six American adults provide care to a family member with serious illness, and digital health interventions have significant potential to support these caregivers' psychological well-being. Relevant to the missions of NIH and NCATS, the long-term goal of this work is to improve digital health interventions for caregivers by determining what tailoring is needed to improve caregivers' ability to use and benefit from these interventions. Towards this goal, this study proposes a mixed-methods approach to assess how caregiving context relates to engagement with and efficacy of an established digital health intervention that delivers cognitive-behavioral therapy for insomnia.

CRITIQUES

Critique 1
Significance: [revoked]
Investigator(s): [revoked]
Innovation: [revoked]
Approach: [revoked]
Environment: [revoked]

Overall Impact: This application will address the question of "What tailoring is necessary and sufficient to achieve optimal engagement with and efficacy of SHUTi for caregivers?" SHUTi (Sleep Healthy Using the Internet) has been tested in a number of trials, but the investigators' recent evidence suggests that caregivers may benefit less than non-caregivers. The revised application is much improved. Previous reviewers had concerns about limited significance, as sleep concerns among caregivers have been well-outlined in the literature, and the lack of clear rationale for studying cancer caregivers compared to dementia caregivers. The investigators have significantly modified the application and now propose two more focused, distinct, aims: Specific Aim 1: Test the association of SHUTi engagement with caregiving context and Specific Aim 2: Efficacy as defined by impact on known cognitive mechanisms such as sleep beliefs, and internalized sleep locus of control. A few minor concerns remain but do little to diminish enthusiasm for the proposed project.

Significance
Strengths
- Insomnia in caregivers is a significant problem given that 1-in-6 American adults are caregivers and are more likely to experience insomnia as a result.
- Internet-delivered interventions overcome access barriers and may be particularly helpful for caregivers who may have difficulty scheduling in-person sessions to address their own health problems.
- Although cognitive behavioral therapy for insomnia has been demonstrated to be efficacious, caregivers may benefit less. Thus, the proposed study will examine the question of how to optimize outcomes for caregivers – what tailoring may be necessary for the program to be engaging and effective for caregivers?

Weaknesses
- Insomnia is only one of a host of common problems stemming from caregiving burden. A focus on insomnia may be a bit narrow, a minor concern.

Investigator(s)
Strengths
- The team is strong and the investigators are experienced and well-poised to successfully complete the proposed work.
- The partnership between CTSA hubs at UVA and the University of Pittsburgh is strong.

Weaknesses
- None noted.

Innovation
Strengths
- The proposed work is appropriately innovative – it uses an existing, previously tested intervention, and tailors it for a particular population with a high need.
- Specific areas of innovation include systematically studying factors that may impact why SHUTi is less effective for caregivers; examining caregiving context as one possible predictor of use/engagement is innovative, for example.

Weaknesses
- None noted.

Approach
Strengths
- Investigators no longer propose to compare cancer vs. dementia caregivers explicitly – instead, they will recruit “high intensity” caregivers (p.64) across care recipient diagnosis.
- The aims are explicitly tied to the theory.
- The two Specific Aims focus on two distinct questions: predictors of engagement and efficacy, respectively. Splitting the aims this way will provide useful information for future iterations of SHUTi as well as other types of caregiver interventions.

Weaknesses
- Specific Aim 1b will assess barriers and motivations for engagement as part of the post intervention questionnaire using open-ended questions. Phone interviews would provide richer data and would be worth the additional time and cost.
- The caregivers who are less engaged in the platform will likely also be the ones who do not complete the follow up assessment. Investigators should consider reaching out to those who have dropped out and do not respond to the assessment invitation either.
- It would have been helpful to see more about the content of SHUTi. What is in each of the six Cores?
- The participants will no longer be exclusively cancer or dementia caregivers, but will the diagnosis of care recipient still be considered part of the context of caregiving? It does not appear that it will.

Environment
Strengths
- UVA and the University of Pittsburgh appear to be supportive environments for the proposed research.

Weaknesses
- None noted.

Study Timeline (Only applicable to applications designated clinical trial on the electronic cover sheet).
Strengths
- The timeline for the proposed work is reasonable. Even in the brief two years of the proposed period, there are three months set aside for development of manuscripts, presentations and planning for an R01.

Weaknesses
- None noted.

Protections for Human Subjects: Acceptable Risks and/or Adequate Protections.

Human Subjects protections seem reasonable.

Data and Safety Monitoring Plan (Applicable for Clinical Trials Only): Acceptable. DSMP is thorough.

Inclusion of Women, Minorities, and Individuals Across the Lifespan: Investigators propose reasonable plans to enroll a diverse sample with respect to gender, race/ethnicity, and age.

- Sex/Gender: Distribution justified scientifically.
- Race/Ethnicity: Distribution justified scientifically.
- For NIH-Defined Phase III trials, Plans for valid design and analysis: Not applicable.
- Inclusion/Exclusion Based on Age: Distribution justified scientifically.

Vertebrate Animals: Not Applicable (No Vertebrate Animals).

Biohazards: Not Applicable (No Biohazards).

Resubmission: Investigators are very responsive to previous critiques and have made a number of significant changes to the application as a result.

Select Agents: Not Applicable (No Select Agents).

Resource Sharing Plans: Acceptable**.** The dissemination plan includes processes for data sharing.

Authentication of Key Biological and/or Chemical Resources: Not Applicable (No Relevant Resources).

Budget and Period of Support: Recommend as Requested.

Critique 2
Significance: [revoked]
Investigator(s): [revoked]
Innovation: [revoked]
Approach: [revoked]
Environment: [revoked]

Overall Impact: This R21 application is a resubmission. In response to the previous review, the proposed Specific Aims and Approach have been significantly modified and overall, changes appear to have been responsive to the previous reviewers' concerns. The purpose is to identify what tailoring of SHUTi is needed for optimal use of this insomnia digital intervention when used by high intensity caregivers. Tailoring SHUTi, an internet based cognitive behavioral therapy for insomnia, is the primary innovation in this study. Overall, the research team appears to have the appropriate expertise and skills. An open label trial, a pre post mixed-methods study is proposed. Some weaknesses are noted including the age range of caregivers, how some variables will be measured, lack of psychometric information on measures, and potential subject burden.

Significance
Strengths

- As noted previously, family caregivers often experience challenges, including difficulty sleeping, which has been well documented.
- Weaknesses noted by the previous reviewers are that sleep disturbances had been studied before in the proposed groups and that the intervention, SHUTi, had been tested in multiple

other studies. However, in this resubmitted application, the purpose is on identifying possible tailoring of SHUTi needed for successful use with high intensity caregivers. Tailoring is a strength of the new, proposed study.
- The focus is now on high-intensity caregiver situations as opposed to the two specific groups described previously, caregivers of cancer and dementia patients.

Weaknesses
- While tailoring may be an appropriate next step based on the findings of this study, it may be that instead of tailoring SHUTi, there are other available interventions for sleep concerns in high intensity caregivers. More information on other digital sleep interventions would be helpful.

Investigator(s)
Strengths
- The Principal Investigator (PI), Kelly Shaffer, PhD, is a clinical psychologist and an Assistant Professor in the School of Medicine, Department of Psychiatry and Neurobehavioral Sciences, at the University of Virginia and is an early-stage investigator. Her research is on caregiver burden and she has a strong publication record. Currently has a mentored Scholar Award/KL2.
- Co-Investigators at UVA: Meghan Mattos, PhD, MSN is an Assistant Professor at UVA School of Nursing and is currently completing a study on SHUTi among older adults with mild cognitive impairment. Lee Ritterband, PhD, is a psychologist and Professor in the School of Medicine, Department of Psychiatry and Neurobehavioral Sciences. He is an internationally recognized researcher on internet health interventions, developer of SHUTi, and has an exceptional funding and publication record. He will provide outstanding mentoring to Dr. Shaffer and the team on this application. Fabian Camacho, MS, Biostatistician Dept. of Public Health Sciences, will provide statistical support.
- Co-investigators at the University of Pittsburgh: Heidi Donovan, PhD, Professor, School of Nursing, whose research focus is on development and testing of theory-guided behavioral interventions for patients with cancer and their family caregivers; and Daniel Buysse, MD, Professor, Department of Psychiatry, whose research experience is in behavioral treatments for insomnia and other sleep problems.
- Overall a strong research team with appropriate expertise and skills. Senior mentor Dr. Ritterband developed SHUTi and is a mentor for both Dr. Shaffer and Mattos on NCATS KL2 awards. Members have expertise in digital health, conducting trials with SHUTi, gerontology, caregivers and statistics.
- All team members will attend twice monthly study meetings.

Weaknesses
- A previous concern was lack of experience with dementia caregivers, but the changes in Aims and Approach make that less of a concern.

Innovation
Strengths
- The primary area of innovation in this project is studying the tailoring of a digital heath intervention. Tailoring is a critical next step in the area of mHealth. Studying what aspects of a particular mHealth intervention should contribute to the tailoring is an important step.

Weaknesses
- While internet delivered cognitive-behavioral therapy for insomnia (CBT-I) may not have been tailored, other CBT digital-based interventions have been delivered and tested for problems other than insomnia. However, it is important to continue studying tailoring of digital interventions both for increased effectiveness for patients and families as well as for potential cost containment.

Approach
Strengths

- The proposed Specific Aims and Approach have been significantly changed from the first submission.
- The overall purpose is to identify what tailoring of SHUTi is needed for optimal use of this insomnia digital intervention when used by high intensity caregivers.
- An open label trial, pre post mixed-methods study is proposed.
- The digital intervention for insomnia used in this application, SHUTi, has been studied the past 15 years via multiple funded projects.
- A recent pilot study of older adults' use of SHUTi found that those identified as caregivers reported less improvement in sleep compared to non-caregivers. This led to the proposed study to identify caregiver context (i.e., user characteristics and environment) and the potential tailoring needs of SHUTi for this group.
- Analysis of open-ended survey responses will include caregiver participants' review.

Weaknesses

- In the description of SHUTi, it is not clear what the six Cores are.
- The small pilot study was with older adults while the proposed study includes all high intensity caregivers over 18 years old. The reason for this change is unclear. As noted in the Inclusion Across the Lifespan section, much of the previous research on SHUTi has been with those 55 and older.
- While the previous proposed study focused on caregivers of cancer and dementia patients, based on the sites described as available at both institutions for subject recruitment, it appears that most subjects will still be caregivers of patients with cancer and dementia.
- Pre-assessment appears to include demographics such as health literacy and quality of life that will be used as covariates in the analysis. However, it is not clear how these will be measured.
- Table 1 lists pre- and post-measures. There appear to be six measures used (Pearlin/four subscales, Caregiver Guilt, Barthel Activities of Daily Living (ADL), Internet Questionnaire, and two sleep scales). Little information is provided about reliability and validity of the measures.
- It states that it takes ~45 minutes to complete the multiple measures, potentially posing subject burden.
- It is not clear who on the team has expertise in qualitative analysis, although Drs. Shaffer, Mattos and Donovan will complete this analysis.

Environment

Strengths

- Both UVA and the University of Pittsburgh have the resources needed for the proposed study. Both are CTSA hubs.
- Both hubs provided strong letters of support.

Weaknesses

- None noted.

Study Timeline (Only applicable to applications designated clinical trial on the electronic cover sheet).

Strengths

- It appears reasonable, including recruiting 10 subjects per month over 10 months for the proposed 100 participants.

Weaknesses

- None noted.

Protections for Human Subjects: Acceptable Risks and/or Adequate Protections.

Data and Safety Monitoring Plan (Applicable for Clinical Trials Only): Acceptable**.**

Overall appropriate plan. There is just some confusion on page 90. Initially it states, "The investigators will assess the relationship of the adverse event as not related, possibly related, or definitely related using standard criteria for clinical trials." However, the criteria that follow are possible, probable and definite.

Inclusion of Women, Minorities, and Individuals Across the Lifespan:
- Sex/Gender: Distribution justified scientifically.
- Race/Ethnicity: Distribution justified scientifically.
- For NIH-Defined Phase III trials, Plans for valid design and analysis: Not applicable.
- Inclusion/Exclusion Based on Age: Distribution justified scientifically.
  Includes caregivers over 18.

Vertebrate Animals: Not Applicable (No Vertebrate Animals).

Biohazards: Not Applicable (No Biohazards).

Resubmission: The investigators appear to be responsive to previous reviewers and have made a number of changes to the application, including changing the population to be studied.

Select Agents: Not Applicable (No Select Agents).

Resource Sharing Plans: Acceptable.

Authentication of Key Biological and/or Chemical Resources: Not Applicable (No Relevant Resources).

Budget and Period of Support: Recommend as Requested.

Critique 3
Significance: [revoked]
Investigator(s): [revoked]
Innovation: [revoked]
Approach: [revoked]
Environment: [revoked]

Overall Impact: This is a resubmission of an R21 application by an early-stage investigator at the University of Virginia School of Medicine. This project aims to examine what level of tailoring may be necessary for evidence-based digital health interventions to achieve optimal engagement with caregivers, and their efficacy. For this project, 100 high-intensity caregivers with insomnia will be recruited to receive access to a digital intervention called SHUTi, which is designed to deliver cognitive behavioral therapy for insomnia in an open-label trial. The study aims to test the association between engagement with the digital tool and caregiving context (including user and environment characteristics) and also test whether the effects of SHUTi on known cognitive mechanisms of change are associated with differences in caregiving-related user and environment characteristics. The focus on ways to inform tailoring of digital interventions is innovative and the examination of digital tools to improve sleep for high intensity caregivers is significant. The PI has previous experience with recruitment of caregivers and has assembled a team with relevant and related expertise (e.g., design of large clinical trials, recruitment of caregivers of different patient populations and insomnia research). It is not entirely clear how the findings will practically inform tailoring of the SHUTi tool, and it is hard to envision how specific user and environmental characteristics of the caregiving context can each be modified or addressed through tailoring of the digital tool. The single arm design focuses on

intervention mechanisms and is appropriate for the study aims and to establish plausibility to support future larger scale efficacy trials. A plan for future directions and follow-up studies is provided. The applicant has been very responsive to previous reviews and has re-designed the approach to provide a solid plan for this and future steps.

Significance
Strengths
- The goal to improve digital health interventions for caregivers is an important one, as caregivers may greatly benefit from supportive tools.
- Additionally, the focus on insomnia and sleep disturbances for high intensity caregivers is clinically significant as evident in scientific literature.
- Determining what tailoring is needed to improve caregivers' ability to benefit from technology-mediate interventions is significant.

Weaknesses
- How the findings will practically inform tailoring is not fully clear. The applicants provide examples of potential modifications (e.g., based on findings, modifying content to be more caregiver-salient or addressing specific caregiving-related barriers), but it is hard to envision how specific user and environmental characteristics of the caregiving context that may impact adoption, can each be modified or addressed through tailoring of the digital tool.

Investigator(s)
Strengths
- The PI has related expertise and experience (has recruited caregivers in psychosocial research, has examined psychiatric symptoms including insomnia experienced by cancer caregivers). Dr. Ritterband serves as co-Investigator, has mentored Dr. Shaffer and has developed the SHUTi program.
- Co-Investigators have significant experience with large clinical trials and designing and delivering interventions for caregivers across multiple disease contexts (e.g., cancer, dementia).

Weaknesses
- None.

Innovation
Strengths
- Focus on ways to inform tailoring of digital interventions is innovative.
- Examining the caregiver context (both in terms of user and environmental characteristics) and how it may correlate to the effectiveness of the digital intervention is innovative.
- Testing a fully automated CBT intervention for insomnia targeting caregivers is a novel focal area.

Weaknesses
- None.

Approach
Strengths
- Single arm design focusing on intervention mechanisms is appropriate to inform future larger scale efficacy trials.
- Previous work shows experience of the team recruiting caregivers, testing SHUTi, delivering CBT for cancer caregivers and developing and evaluating internet-based insomnia interventions.
- Dr. Ritterband developed the Model for Internet Interventions, which informed the development of SHUTi.
- Variables and scales to measure the intervention are well defined and described. Most are established scales, although for lesser-known ones validity and reliability are not reported.

- The investigators will make use of both quantitative and qualitative methods to assess user reactions to the digital tool and identify potential barriers to use.

Weaknesses

- While it is important to have a theoretical framework for the study, the Model for Internet Interventions is based on hypotheses as to what factors may relate or inform usage and adoption of an intervention. It is not clear if this model has been validated empirically.

Environment

Strengths

- UVA is an appropriate environment for the proposed work, with various clinical sites that can facilitate recruitment such as the UVA Cancer Center and the UVA Memory and Aging Care Clinic. The University of Pittsburgh as a collaborating site is also well-suited for the study.
- The Center for Behavioral Health and Technology at UVA and the Center for Caregiving Research at the University of Pittsburgh, as well as the two CTSA hubs provide excellent resources for the proposed work.

Weaknesses

- None

Study Timeline (Only applicable to applications designated clinical trial on the electronic cover sheet).

Strengths

- Timeline is appropriate and shows feasibility, with calculations that indicate recruiting ten caregivers per month to meet their recruitment goal.

Weaknesses

- The applicant indicates that in the first three months after project start date the IRB application will be submitted, reviewed and approval will be granted. Typically, IRB approval is required prior to initiating the project (given that the timeline is two years, which is the duration of the R21 project). Ideally, the timeline starts at point zero where IRB approval has already been obtained.

Protections for Human Subjects: Acceptable Risks and/or Adequate Protections.
Risks and benefits have been identified and discussed.

Data and Safety Monitoring Plan (Applicable for Clinical Trials Only): Acceptable**.**
There is a detailed data and safety monitoring plan in place and concerns and adverse events have been clearly identified. While there is no need for a monitoring board, it might be good to have someone in addition to the PI in charge of monitoring, addressing, and resolving all issues.

Inclusion of Women, Minorities, and Individuals Across the Lifespan:
Participants over the age of 18 will be recruited (as appropriately justified) with no upper age limit. Race/ ethnicity and gender inclusion are appropriately described.

- Sex/Gender: Distribution justified scientifically.
- Race/Ethnicity: Distribution justified scientifically.
- For NIH-Defined Phase III trials, Plans for valid design and analysis: Not applicable.
- Inclusion/Exclusion Based on Age: Distribution justified scientifically.

Vertebrate Animals: Not Applicable (No Vertebrate Animals).

Biohazards: Not Applicable (No Biohazards).

Resubmission: The applicant has been very responsive to reviewers' comments. The application has shifted focus from comparison between disease groups to high intensity caregivers. Additionally, the

aims are no longer interdependent and significant considerations have been introduced to assess caregiver context.

Select Agents: Not Applicable (No Select Agents).

Resource Sharing Plans: Acceptable**.**
Extensive plan for dissemination; de-identified data sets will also be shared.

Authentication of Key Biological and/or Chemical Resources: Not Applicable (No Relevant Resources).

Budget and Period of Support: Recommend as Requested.

THE FOLLOWING SECTIONS WERE PREPARED BY THE SCIENTIFIC REVIEW OFFICER TO SUMMARIZE THE OUTCOME OF DISCUSSIONS OF THE REVIEW COMMITTEE, OR REVIEWERS' WRITTEN CRITIQUES, ON THE FOLLOWING ISSUES:

PROTECTION OF HUMAN SUBJECTS: ACCEPTABLE

INCLUSION OF WOMEN PLAN: ACCEPTABLE

INCLUSION OF MINORITIES PLAN: ACCEPTABLE

INCLUSION ACROSS THE LIFESPAN: ACCEPTABLE

VERTEBRATE ANIMALS: NOT APPLICABLE

BIOHAZARD COMMENT: NOT APPLICABLE

SELECT AGENTS: NOT APPLICABLE

RESOURCE SHARING PLANS: ACCEPTABLE

AUTHENTICATION OF KEY BIOLOGICAL AND CHEMICAL RESOURCES: NOT APPLICABLE

COMMITTEE BUDGET RECOMMENDATIONS: The budget is recommended as requested.

Footnotes for 1 R21 TR003522-01A1; PI Name: Shaffer, Kelly McLean

NIH has modified its policy regarding the receipt of resubmissions (amended applications).See Guide Notice NOT-OD-18-197 at https://grants.nih.gov/grants/guide/notice-files/NOT-OD-18-197.html. The impact/priority score is calculated after discussion of an application by averaging the overall scores (1-9) given by all voting reviewers on the committee and multiplying by 10. The criterion scores are submitted prior to the meeting by the individual reviewers assigned to an application, and are not discussed specifically at the review meeting or calculated into the overall impact score. Some applications also receive a percentile ranking. For details on the review process, see http://grants.nih.gov/grants/peer_review_process.htm#scoring.
