# Supplementary material for: Single-Group Trial of an Internet-Delivered Insomnia Intervention Among Higher-Intensity Family Caregivers: Rationale and Protocol for a Mixed Methods Study
Source: JMIR Res Protoc. 2022 Jan 12;11(1):e34792. doi: 10.2196/34792 (PMC8792774; doi:10.2196/34792)
Supplement: Multimedia Appendix 2 [file resprot_v11i1e34792_app2.pdf]

RESPONSE TO NCATS LEADERSHIP CONCERNS

We thank the NCATS leadership team for considering our proposal, "*Optimizing efficiency and impact of digital health interventions for caregivers: A mixed methods approach*" (R21 TR003522-01). Here, we provide a detailed response to two primary concerns raised about the proposal: (1) assessment burden and (2) the cognitive-behavioral focus of the targeted intervention. We have carefully considered these concerns. In response, we have shortened the total assessment burden for the typical participant by about 180 items, or an estimated 40 minutes overall. We have also outlined the tenets of CBT-I to better indicate how they would benefit this population. Following our detailed response, we provide a one-page formal response to reviewers.

Assessment Burden

We appreciate the concerns regarding potential burden of the research assessments. We have carefully reviewed our assessment batteries to balance scientific rigor and participant safety against participant time and burden. It is important to note that many of these assessments are <u>for research purposes only</u>. Should SHUTi be implemented as part of routine care, assessments would be limited to minimal screening (to ensure caregivers with potential medical and/or psychiatric counterindications are not provided the program) and sleep diaries, which are a component of intervention tailoring.

Phone screening: This screening includes a brief medical history review and structured clinical interview to ensure that caregivers with potential counterindications to cognitive-behavioral therapy for insomnia are not enrolled. By removing clinical assessments not strictly assessing contraindications, <u>we reduced the estimated time for completion of this screening by 10 minutes (25%) to approximately 30 minutes</u>.

Baseline questionnaire assessment: This assessment battery includes constructs fundamental to our proposed aims, and uses brief, validated scales to reliably assess these constructs. By carefully reducing included constructs and identifying shorter validated scales for included constructs where possible, <u>we reduced the estimated completion time for the baseline assessment by 15 minutes (33%) to approximately 30 minutes</u>.

Post-assessment questionnaires for SHUTi users: SHUTi users will complete an online questionnaire battery of both validated self-report scales and free-response items assessing constructs fundamental to our proposed aims and their evaluation of SHUTi. We have added 3 items to assess participants' use and interest in wearable activity monitors for reducing assessment burden in future trials. By carefully reducing included constructs and identifying shorter validated scales for included constructs where possible, <u>we reduced the estimated completion time for the baseline assessment by 15 minutes (33%) to approximately 30 minutes</u>.

We have also sought to balance assessment burden with capturing actionable feedback from caregivers regarding potential tailoring needs. Thus, we have retained the battery of open-ended items that assess this information. Based on feedback from reviewers, we are also willing to include optional phone interviews during the synthesized member checking phase of the study with caregivers who are interested and willing to provide more in-depth information about their experiences using SHUTi as a high-intensity caregiver.

Post-assessment questionnaires for SHUTi non-users: SHUTi non-users will complete a brief online questionnaire battery of both multiple choice and free-response items assessing constructs fundamental to our proposed aims and their barriers to initiating SHUTi. We have added 3 items to assess participants' use and interest in wearable activity monitors for reducing assessment burden in future trials. By carefully reducing included constructs and identifying shorter validated scales for included constructs where possible, <u>we reduced the estimated completion time for the baseline assessment by 10 minutes (50%) to approximately 10 minutes</u>.

Passive sensing to reduce assessment burden: We continue to consider the use of passive sensing by wearable devices (e.g., fitbit) or ambient sensors (e.g., RFID) to reduce or eliminate sleep diary completion as part of SHUTi. We are working to build out these technical capabilities and test the impact on intervention engagement and efficacy, given it is unknown whether the completion of sleep diaries serves as an engagement and/or cognitive intervention component. While these studies are ongoing, we added items to our assessment battery capturing caregivers' ownership and use of wearable devices, as well as their level of

interest in using passive sensors to reduce daily diary commitment. Our follow-up studies will be informed by the joint findings of our ongoing work to develop technical capabilities to use passive sensing with SHUTi with our direct assessment of caregiver interest in these capabilities in the present proposal.

Cognitive-Behavioral Focus of Intervention

We also appreciate the consideration of factors outside of maladaptive cognitions and behaviors related to caregivers' insomnia. Here, we provide a more complete description of the SHUTi intervention to establish that this intervention addresses the broader, complex nature of insomnia beyond thoughts and behavior. We also emphasize how our primary aims of the proposal will help us pinpoint where a more targeted focus on environmental and/or caregiving factors may be necessary for high-intensity caregivers to maximally benefit from this intervention.

SHUTi program: SHUTi is a self-guided, fully automated, interactive, and tailored Internet-based program modeled on the primary tenants of face-to-face cognitive-behavioral therapy for insomnia (CBT-I; sleep restriction, stimulus control, cognitive restructuring, sleep hygiene, relapse prevention). CBT-I is the first-line recommended treatment for insomnia.[1] The primary model of behavior change for SHUTi is based on Social Cognitive Theory (SCT).[2] SCT, the most widely used model of behavior change in the Internet intervention literature,[3] explains behavior change through the dynamic interaction of behavior, personal factors (e.g., cognitions), and the environment. SHUTi content is metered out over time through six "Cores." As demonstrated in the table of SHUTi content, the intervention addresses insomnia by targeting modifiable insomnia-perpetuating cognitive and behavioral factors, it also clearly addresses how non-modifiable life events influence insomnia.

#	Name	Content
1	Overview	Insomnia defined, types, prevalence, risk factors, and impact (i.e., daytime fatigue, psychological well-being, physical health, economic cost); Setting treatment goals; Treatment overview, appropriateness, and effectiveness
2	Sleep Behavior 1	Explanation of poor sleep habits; Situational / chronic sleep difficulties; Cycle of chronic insomnia; Introduction of sleep restriction; Explanation of sleep efficiency (SE); Instruction on adjustments of sleep window based on SE
3	Sleep Behavior 2	Introduction of stimulus control (i.e., going to bed when sleepy, leaving bed if unable to sleep, regular sleep schedule, using bed for sleep only, no napping)
5	Sleep Thoughts	Relationship between thinking patterns and emotions; Contributions of thought patterns to sleeplessness; Cognitive restructuring; Keeping realistic expectations; Revising misconceptions about insomnia; Eliminating catastrophizing; Reducing sleep emphasis; Developing tolerance for sleep loss effects; Dealing with setbacks
4	Sleep Education	Sleep hygiene guidelines; avoiding stimulants; effects of diet, environment, and exercise
6	Problem Prevention	Relapse prevention techniques; Considering therapeutic gains; Review of sleep behavior techniques; Sleep medication information; Maintaining program techniques

Current proposal: The central question of our proposal directly addresses the tension highlighted by NCATS leadership: *What tailoring is necessary and sufficient to achieve optimal engagement with and efficacy of SHUTi for caregivers?* On one hand, SHUTi has demonstrated robustly positive effects among medically- and psychiatrically-diverse samples in multiple clinical trials, and our preliminary evidence suggests caregivers generally reported comparable satisfaction, fit, and usability of SHUTi compared to non-caregivers. On the other hand, there is reason to believe that caregiving has unique psychological and environmental factors that would affect use and impact of SHUTi, and our preliminary evidence suggests that caregivers did not report as significant treatment benefits as non-caregivers. By directly assessing to what extent caregiving user- and environmental characteristics are related to SHUTi uptake, engagement, and efficacy, we will establish to what extent – and how – caregiving-specific tailoring is needed to maximize caregivers' benefit from SHUTi. Moreover, our analyses will determine the exent to which these findings extend to other evidence-based digital health interventions for family caregivers.

INTRODUCTION AND RESPONSE TO SUMMARY STATEMENT: R21 TR003522-01

We are grateful for reviewers' supportive comments, which commended our "important topic," and "strong and experienced team of investigators." Our modifications in this A1 application were described as "highly responsive," with a "re-designed approach to provide a solid plan for this [study] and future steps." We address the constructive critiques of our reviewers below:

Reviewers 1 & 3: We are grateful for the strong support of R1 and R3. **R1:** We agree that insomnia is one of a host of psychological concerns of caregivers, and clarify that information gained by understanding what tailoring is necessary and sufficient to optimize the usability and efficacy of our online insomnia program for caregivers will also be pertinent to the tailoring of other evidence-based digital health programs for caregivers. Although we have retained open-ended questions to assess the entire sample for feasibility, we will include phone interviews with a subset of caregivers, who indicate that they are willing and interested, during the synthesized member checking process, per reviewer suggestion, to elicit further detail about potential tailoring needs. As suggested, we will also include a robust protocol for reaching non-completers to limit asymmetric attrition across levels of engagement. **R3:** We clarify how findings will practically inform tailoring: not by altering the caregiving context, but instead, by altering the content or delivery of the intervention to be more accessible or acceptable given the context. Examples include breaking Cores into smaller segments, or using more audio to allow multi-tasking. Our research team has published multiple papers validating elements of the Model for Internet Interventions.[4–7]

Reviewer 2: We appreciate the critiques of R2. As in response to R1, we emphasize the importance of information gained from this proposal regarding tailoring to optimize usability and efficacy of SHUTi also being pertinent to the tailoring of other digital health and insomnia programs for caregivers, enhancing the translational significance of this work. We also clarify that, although the latest R01-level clinical trial of SHUTi exclusively recruited adults 55 and older, multiple clinical trials of SHUTi have recruited adults of all ages and demonstrated positive effect of the program. Alzheimer's disease/dementia and cancer are among the top 5 most common main care recipient problems (accounting for 11% and 6% of caregivers, respectively).[8] Thus, we expect caregivers for people with dementia or cancer will be highly represented in our sample. However, our recruitment methods (including research registry outreach, in-clinic recruitment, and online national recruitment methods) will target high-intensity caregivers across caregiving contexts. In particular, our primary recruitment sources – the National Rehabilitation Research and Training Center on Family Support and the University Center for Social and Urban Research Survey Research Program research registry – are both agnostic to caregiving condition. We will also work with our CTSA recruitment support teams to ensure a distribution of caregiving contexts in our study. Regarding study measures, all study have been selected for their robust validation and use in our prior trials with caregivers. The reviewer raises an important consideration related to assessment burden for this population. We have carefully reviewed our batteries to use shorter validated scales where possible and remove any assessments that are not strictly necessary for completing and interpreting proposed study aims. We will emphasize with participants that they may take breaks in completing the questionnaire battery, returning to complete the surveys where they left off via features embedded in our online survey tools. Lastly, we document here Dr. Shaffer's expertise in qualitative research methods. She received qualitative research training as part of her NCI T32 Fellowship in Psycho-oncology Research Methods. She has led as Primary Investigator four qualitative studies, two specifically with family caregivers. She has led the qualitative coding analyses and write-up for four manuscripts across three separate datasets (Shaffer et al., 2019, Psycho-Oncology; Shaffer et al., 2019, Oncology Nursing Forum; Shaffer et al., preprint, doi:10.31219/osf.io/q4enf – under review, Journal of Psychosocial Oncology; Shaffer et al., 2021, Supportive Care in Cancer), and she provides qualitative expertise as a Co-Investigator (NCI R37CA248434) and consultant (NCI SBIR 75N91020C00045-0-9999-1) on other current NIH-funded projects.

In addition, both **R1** and **R2** asked for more detail about the six SHUTi Cores: we clarify that Cores act as an online analog for the weekly sessions typically used when delivering CBT for insomnia in a face-to-face format, covering content on: (1) program overview, (2 & 3) behavioral insomnia treatment strategies, (4) cognitive insomnia treatment strategies, (5) sleep hygiene education, and (6) relapse prevention. We direct reviewers to Thorndike et al., 2008 for complete program details.

In summary, we are grateful for the strong support of the reviewers. By addressing reviewers' critiques, the rigor of our proposed methods and significance of study findings are clarified and enhanced. As such, this proposal has even greater potential to address the central research question on tailoring interventions for caregivers that reviewers described as "important," "significant," and "innovative."

References

1. Schutte-Rodin S, Broch L, Buysse D, Dorsey C, Sateia M. Clinical guideline for the evaluation and management of chronic insomnia in adults. *Journal of Clinical Sleep Medicine*. 2008;4:487-504.

2. Bandura A. *Social Foundations of Thought and Action: A Social Cognitive Theory*. Prentice-Hall; 1986.

3. Webb TL, Joseph J, Yardley L, Michie S. Using the internet to promote health behavior change: A systematic review and meta-analysis of the impact of theoretical basis, use of behavior change techniques, and mode of delivery on efficacy. *Journal of Medical Internet Research*. 2010;12(1):e4.

4. Ritterband LM, Borowitz SM, Cox DJ, et al. Using the internet to provide information prescriptions. *Pediatrics*. 2005;116(5):e643-e647.

5. Ritterband LM, Cox DJ, Gordon T, et al. Examining the added value of audio, graphics, and interactivity in an Internet intervention for pediatric encopresis. *Child Health Care*. 2006;35(1):47-59.

6. Magee JC, Ritterband LM, Thorndike FP, Cox DJ, Borowitz SM. Exploring the relationship between parental worry about their children's health and usage of an internet intervention for pediatric encopresis. *Journal of Pediatric Psychology*. 2009;34(5):530-538.

7. Chow PI, Ingersoll KS, Thorndike FP, et al. Cognitive mechanisms of sleep outcomes in a randomized clinical trial of internet-based cognitive behavioral therapy for insomnia. *Sleep Medicine*. 2018;47:77–85.

8. National Alliance for Caregiving. *Caregiving in the US*. AARP Public Policy Institute; 2020.

9. Shaffer K, Ingersoll K, Chow P, et al. Timing and tailoring of internet-based cognitive-behavioral treatment for insomnia for cancer survivors: A qualitative study. *Psycho-Oncology*. 2019;28(9):1934-1937.

10. Shaffer KM, Benvengo S, Zaleta AK, et al. Feasibility and Acceptability of Distress Screening for Family Caregivers at a Cancer Surgery Center. *Oncology Nursing Forum*. 2019;46(2):159-169.

11. Shaffer K, Kennedy E, Glazer J, et al. Including Partners in Discussions of Sexual Side Effects from Breast Cancer: A Qualitative Study of Survivors, Partners, and Providers. Preprint 2021. https://osf.io/q4enf

12. Shaffer K, Kennedy E, Glazer J, et al. Addressing sexual concerns of female breast cancer survivors and partners: A qualitative study of survivors, partners, and oncology providers about Internet intervention preferences. *Supportive Care in Cancer*. Published online. doi:10.1007/s00520-021-06302-w

13. Thorndike FP, Saylor DK, Bailey ET, Gonder-Frederick L, Morin CM, Ritterband LM. Development and perceived utility and impact of an Internet intervention for insomnia. *E-Journal of Applied Psychology: Clinical and Social Issues*. 2008;4(2):32-42.
